# The Features of Genetic Prion Diseases Based on Chinese Surveillance Program

**DOI:** 10.1371/journal.pone.0139552

**Published:** 2015-10-21

**Authors:** Qi Shi, Wei Zhou, Cao Chen, Bao-Yun Zhang, Kang Xiao, Xiu-Chun Zhang, Xiao-Jing Shen, Qing Li, Li-Quan Deng, Jian-Hua Dong, Wen-Qing Lin, Pu Huang, Wei-Jia Jiang, Jie Lv, Jun Han, Xiao-Ping Dong

**Affiliations:** 1 State Key Laboratory for Infectious Disease Prevention and Control, National Institute for Viral Disease Control and Prevention, Chinese Center for Disease Control and Prevention, Beijing, China; 2 Collaborative Innovation Center Diagnosis and Treatment of Infectious Diseases, Zhejiang University, Hangzhou, China; 3 Beijing Centers for Disease Control and Prevention, Dongcheng District, Beijing, China; 4 Henan Provincial Center for Disease Control and Prevention, Zhengzhou, China; 5 An hui Provincial Center for Disease Control and Prevention, Hefei, China; 6 Department of infectious disease control and Prevention, Jilin Provincial Center for Disease Control and Prevention, Changchun, China; 7 Shaanxi Provincial Center for Disease Control and Prevention, Xi’an, China; 8 Institute for Infectious Disease Control and Prevention, Guangdong provincial Center for Disease Control and Prevention, Dashing Town, Panyu District, Guangzhou, China; 9 Deptartment of Acute Communicable Disease Control & Prevention, Shanghai Municipal Center for Disease Control and Prevention, Shanghai, China; 10 Institute of Infectious Diseases Prevention and Control, GuiZhou province Center for Disease Control and Prevention, Guiyang, GuiZhou, China; 11 Tianjin Centers for Diseases Control and Prevention, Hua Yue Street, Hedong District, Tianjin, China; 12 Chinese Academy of Sciences Key Laboratory of Pathogenic Microbiology and Immunology, Institute of Microbiology, Chinese Academy of Sciences, Beijing, China; Deutsches Zentrum für Neurodegenerative Erkrankungen e.V., GERMANY

## Abstract

**Objective:**

To identify the features of Chinese genetic prion diseases.

**Methods:**

Suspected Creutzfeldt-Jakob disease (CJD) cases that were reported under CJD surveillance were diagnosed and subtyped using the diagnostic criteria issued by the WHO. The general information concerning the patient, their clinical, MRI and EEG data, and the results of CSF 14-3-3 and *PRNP* sequencing were carefully collected from the database of the national CJD surveillance program and analyzed using the *SPSS 11*.*5* statistical software program.

**Results:**

Since 2006, 69 patients were diagnosed with genetic prion diseases and as having 15 different mutations. The median age of the 69 patients at disease onset was 53.5 years, varying from 19 to 80 years. The majority of patients displaying clinical symptoms were in the 50–59 years of age. FFI, T188K gCJD and E200K were the three most common subtypes. The disease appeared in the family histories of 43.48% of the patients. The clinical manifestations varied considerably among the various diseases. Patients who carried mutations in the N-terminus displayed a younger age of onset, were CSF 14-3-3 negative, had a family history of the condition, and experienced a longer duration of the condition. The clinical courses of T188K were significantly shorter than those of FFI and E200K gCJD, while the symptoms in the FFI group appeared at a younger age and for a longer duration. Moreover, the time intervals between the initial neurologist visit to the final diagnosis were similar among patients with FFI, T188K gCJD, E200K gCJD and other diseases.

**Conclusion:**

The features of Chinese genetic prion diseases are different from those seen in Europe and other Asian countries.

## Introduction

Human prion diseases are a group of rare, fatal and transmissible neurodegenerative diseases. They are etiologically classified as sporadic, inherited or acquired and include Creutzfeldt-Jakob disease (CJD), Kuru, Gerstmann—Sträussler—Scheinker syndrome (GSS) and fatal familial insomnia (FFI) [1]. CJD has an incidence of 1 or 2 cases per million per year and manifests as rapidly progressive dementia, cerebellar ataxia, myoclonus and behavioral changes. Generally, approximately 85% of all CJD cases are sporadic, 10–15% of the cases are inherited and less than 1% of the cases are infectious [2]. Prion diseases are characterized by an accumulation of an abnormally folded isoform (PrP^Sc^) of the host encoded cellular prion protein (PrP^C^) [3]. In humans, the PrP protein is encoded by the *PRNP* gene, which is located on chromosome 20. Currently, more than 55 dominant mutations in the *PRNP* gene that are directly associated with human genetic prion diseases have been described [4]. In neurology, human genetic prion diseases include genetic CJD (gCJD), GSS and FFI.

The clinical manifestations and neuropathological abnormalities of human genetic prion diseases may not only differ considerably from those of sporadic CJD (sCJD) but also vary largely among themselves depending on the various genotypes [5]. In addition to the routine diagnostic elements that are utilized for CJD, the definitive diagnosis of the human genetic prion disease requires genetic evidence of a mutation in *PRNP*. Since 2006, China has conducted a CJD surveillance program that focuses primarily on 12 provinces. As part of this CJD surveillance, all reported suspected cases in the China National Surveillance Program for CJD have undergone *PRNP* sequencing. Through Sept 2014, 69 different genetic prion diseases have been identified and diagnosed using the fixed genetic data, thereby covering 15 different diseases.

In this study, we present and compare the demographic, epidemiological, clinical and laboratory data of these 69 patients. The majority of patients who display clinical symptoms are 50–59 years or 40–49 years of age. FFI, T188K and E200K gCJD were the most frequently identified genetic prion diseases in China. The time intervals between disease onset and the first neurologist visit and the end of the disease among the different genetic prion diseases, and particularly of FFI, T188K and E200K gCJD, were analyzed. The time interval between the neurologist visit and the final diagnosis and the factors that potentially influence this interval, including the disease subtype, surveillance year, age at onset and permanent residence of the patients, were also evaluated.

## Materials and Methods

### Ethics Statement

The use of patient information stored in the China CJD Surveillance System has been approved by the Research Ethics Committee of the National Institute for Viral Disease Control and Prevention of the China CDC. The written informed content of each suspected case was obtained either through a family member or a relative of the patient, according to the requirement of CJD surveillance.

### Constitution of the national CJD surveillance system

Chinese national CJD surveillance formally began in 2006 under the leadership of the China CDC. It consisted of 12 provincial CDCs and 15 hospitals, which are located in Beijing, Shanghai, Tianjin, Chongqing, Jilin, Shaanxi, Hubei, Guangdong, Guizhou, Anhui, Henan and Xinjiang [6]. These sentinel hospitals are large university hospitals that employ experienced neurologists. The surveillance program was approved by the Ethical Review Committee of the China CDC. Using surveillance documents, the clinical data and specimens of the suspected patients were collected by a clinician from the hospitals. Meanwhile, the staff of the provincial CDCs collected epidemiological data. The collected data and samples were transferred to the national reference laboratory for human prion disease in China CDC, for laboratory tests and a final diagnosis [7].

### Case definition

The suspected CJD cases that were reported from CJD surveillance were diagnosed and subtyped using the diagnostic criteria of the China CDC, which was designed using the diagnostic criteria for CJD from the WHO [8]. Since 2010, the addition of a high signal in the caudate/putamen using an MRI brain scan, according to the renewed version of diagnostic requirements that was issued by the WHO [9]. The clinical and epidemiological data of the suspected patients were collected using designed questionnaires. The clinical data included the general information of the patient, main clinical manifestations, the major symptom and results of clinical examinations (e.g., MRI, EEG and routine CSF biochemistry). The epidemiological data included the location of residence, family history, anamnesis (e.g., surgical or neurosurgical history, organ transplantation, blood donation and transfusion, use of extracts of pituitary or other blood products), and profession. The laboratory tests, including a neuropathological assay, immunohistochemistry (IHC) analysis of PrP^Sc^ in brain tissues, Western blots for PrP^Sc^ in brain tissues and for 14-3-3 in the CSF, and *PRNP* PCR and sequencing were performed using established protocols, as previously described [10,11]. The final diagnosis was made by an expert board of neurologists, neuropathologists, epidemiologists and laboratory staff.

### Study population and data collection

All 69 patients with a diagnosed genetic human prion disease, including gCJD, FFI and GSS, reported via the China CJD surveillance program between Jan 2006 and Sept 2014 were enrolled in this study. The general information concerning the patient, their clinical data, MRI and EEG data, and the results of CSF 14-3-3 and *PRNP* sequencing were carefully collected from the database of the national CJD surveillance program and analyzed using *SPSS 11*.*5* statistical software program. The geographical distribution of the patients was calculated using the provinces in which they permanently lived. EEG abnormalities were recorded only in the event that periodic sharp wave complexes (PSWC) were observed. MRI abnormalities were recorded in the event of a high signal in the caudate/putamen. The interval between the onset of the disease and the first neurologist visit was calculated according to the appearance of neurological or mental symptoms during the first hospital visit with neurologists. The interval between disease onset and diagnosis was calculated using a method described by the CJD surveillance center.

### Laboratory tests

Blood, CSF and brain tissue of suspected patients were collected by the sentinel hospital. All samples were transported to the national reference laboratory for human prion disease in CCDC. Peripheral blood leukocytes were used for sequencing analysis of *PRNP* and of polymorphisms of codon 129 using an automatic genetic analyzer (ABI3130XL). CSF samples were obtained by a routine lumbar puncture and analyzed using a Western blot to detect protein 14-3-3. Brain tissue samples were obtained during an autopsy or biopsy and analyzed using neuropathologic assays and for the detection of PrP^Sc^ using immunohistochemistry and/or Western blot. The standard operation procedure (SOP) of each test has been documented in the CJD surveillance program, which has been previously described [6,7].

### Statistical assays

Statistical analyses were performed using the *SPSS 11*.*5* statistical software program.

## Results

### The constitutions and distributions of Chinese genetic prion diseases

Between Jan 2006 and Sept 2014, a total of 1670 suspected genetic prion disease cases were reported to the CJD surveillance system of China. Among these, 721 sCJD cases were diagnosed, including two definitive, 538 probable and 181 possible cases. Of these, 69 cases with various genetic prion diseases were identified and diagnosed. Five of these cases underwent brain autopsies upon death for neuropathological examination. The blood samples of all suspected CJD cases were collected and *PRNP* sequencing assays were performed. Among the 69 cases of genetic prion diseases, 15 different types of human genetic prion diseases were identified, including D178N FFI (27 cases), T188K gCJD (16 cases), E200K gCJD (9 cases, 13%), P102L GSS (3 cases), G114V gCJD (2 cases), E196A gCJD (2 cases), R208H gCJD (2 cases), R148H gCJD (1 case), V180I gCJD (1 case), T183A gCJD (1 case), E196K gCJD (1 case), E200A gCJD (1 case), V203I gCJD (1 case), as well as gCJD with 1-extra-octarepeat (1 OR) insertion (1 case) and 7-extra-octarepeat (7 ORs) insertion (1 case). Three types of these diseases, FFI (39%), T188K gCJD (23%) and E200K gCJD (13%), were predominant.

**Fig 1:**
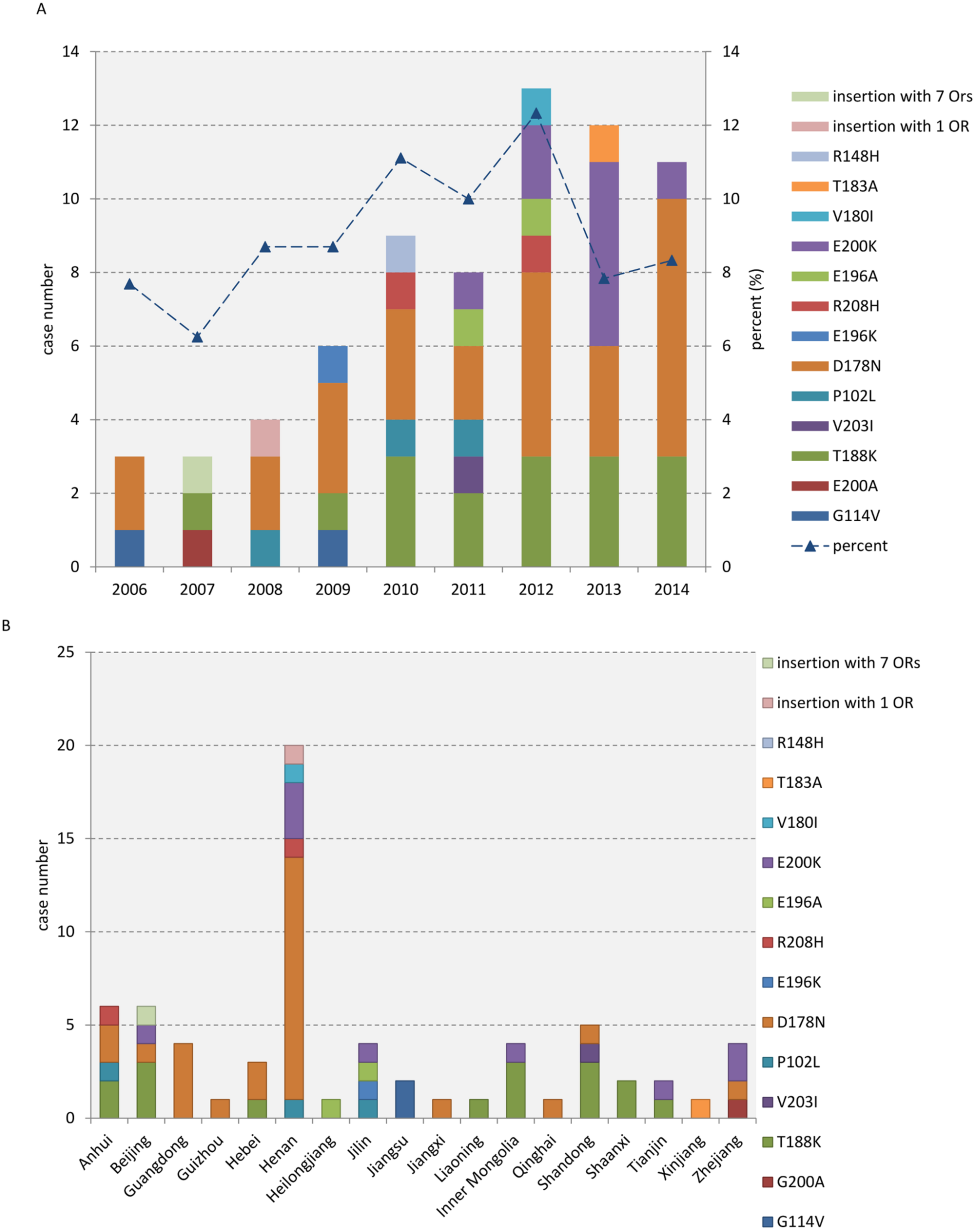
The distributions of the subtypes of genetic prion diseases. A. Surveillance year distribution. The rates of genetic prion diseases among all diagnosed human prion diseases are shown by a blue triangle and a dotted line. B. Geographic distribution. The residences of the patients are given by province.

The number of diagnosed cases of genetic prion diseases per year are shown in Fig 1A. More cases have been identified since 2010, which is concomitant with the increase in reported suspected CJD cases since 2010. Upon calculating the portion of all CJD cases that were genetic prion diseases, we identified that the rate of genetic prion diseases varied from 6.25% to 12.33%, with the average rate being 9.0 ± 1.77%. FFI cases were identified in every surveillance year, with the exception of 2007. To understand the geographical distribution of Chinese patients with genetic prion diseases, the case numbers were sub-counted based upon their permanent residences in the provinces. The patients were observed to have lived in 18 different provinces. Among these provinces, the three with the highest density of cases included Henan (20 cases), Anhui (6 cases) and Beijing (6 cases) (Fig 1B). A previous epidemiological survey verified that, in addition to the two FFI cases (an uncle and a niece) [12,13,14] and the two G114V gCJD cases (an aunt and niece) [15,16], no further genetic relationships could be identified.

### The demographic and clinical features of Chinese genetic prion diseases

**Fig 2:**
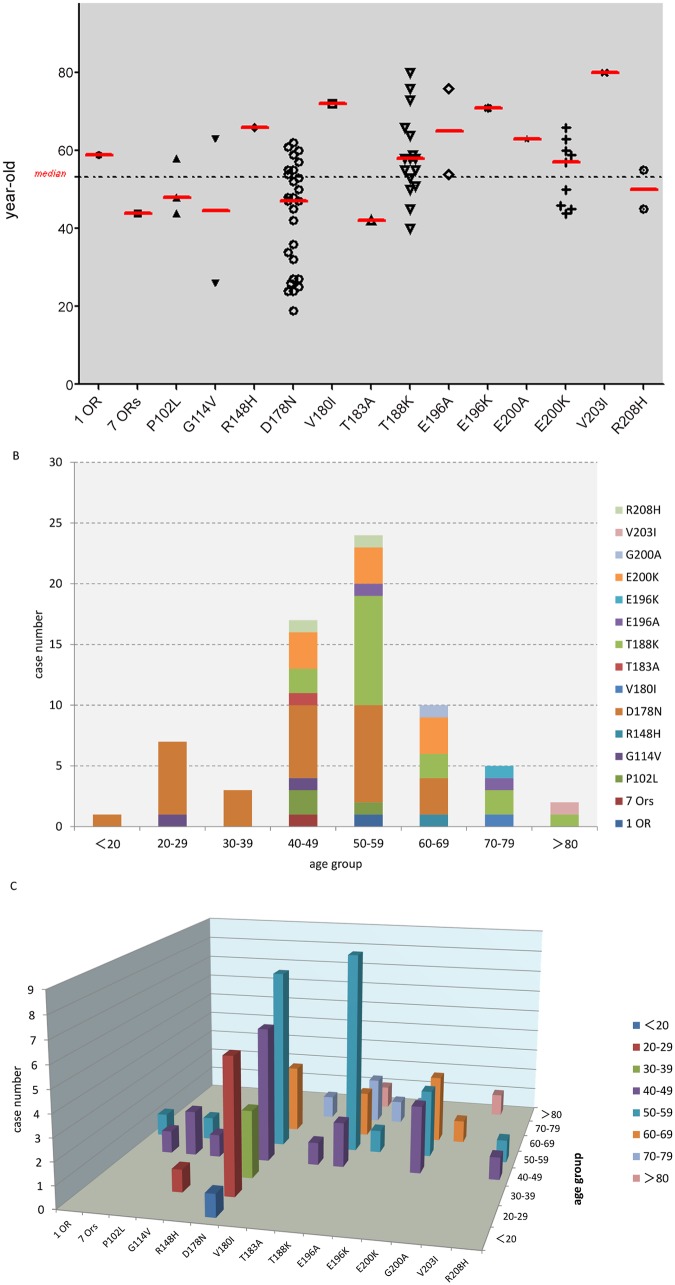
The age of onset of patients of various genetic prion diseases. A. Distribution of the age of onset based upon the types of disease. The median onset age of all 69 patients is indicated by a dotted line. The median onset age of each disease is illustrated with a short, red bar. B. Distribution of the age of onset based upon decade of life. C. Distribution of the age of onset based upon the disease type and decade of life.

Among the 69 cases, 68 patients were Han-Chinese and one was Miao-Chinese. All patients were Met/Met homozygotes at codon 129 in the *PRNP* gene. The gender ratio of male/female was 1/1.09 (33/36). The occupations and education levels of the patients varied largely, with no disease relationship. The median age of onset among the 69 patients was 53.5 years of age and varied between 19 and 80 years of age. Among the patients, the average age of onset for 7-ORs, P102L, G114V, D178N, T183A and R208H was below the median onset age of onset of patients with a genetic prion disease. The average age of onset for the remaining types of disease are shown in Fig 2A. An age-group analysis revealed that the majority of the patients were between 50–59 (24; 34.78%), 40–49 (16; 23.19%) and 60–69 (10; 14.49%) years of age (Fig 2B). Disease-age distributions are illustrated in Fig 2C. Among patients who carried mutations in the N-terminal segments, such as insertions of extra octarepeats, those in the P102L and G114V groups demonstrated an earlier clinical manifestation than did the other groups. For example, the patient with a 7-extra-octarepeat insertion presented clinical signs of the disease much earlier than did the case with a 1-extra-octarepeat insertion. The age of onset in the D178N FFI cases was distributed widely, with no FFI case being identified above 70 years of age. Conversely, the age of onset of the T188K and E200K gCJD cases was older, with no case being identified before 40 years of age. In addition, other gCJD cases with mutations in the C-terminal region, such as V180I, E196A, E196K, E200A and V203I, demonstrated clinical manifestations at older ages.

The clinical symptoms and signs of the Chinese genetic prion diseases varied considerably. Based upon the major neurological symptoms of sporadic CJD, including dementia, myoclonus, visual or cerebella disturbance, pyramidal or extrapyramidal dysfunction, akinetic mutism, the presence and/or rate of various genetic prion diseases are summarized in Table 1. Approximately 70% of FFI patients were recorded as having dementia during the clinical course of their diseases and approximately half of them presented with myoclonus, visual or cerebella disturbance, and pyramidal or extrapyramidal dysfunction. Only a small portion (11.11%) of FFI patients was recorded as having akinetic mutism at the late stage of the disease. Insomnia and hypersomnia were the most common symptoms in approximately 70% of FFI cases. Along with the progression of the disease, sleep disturbances appeared in all FFI cases and persisted through the entirety of the clinical course. Some of the progressive sympathetic symptoms, including excessive sweating, salivation, minor evening pyrexia and weight loss, were noticed in most cases [12, 13]. Three of the FFI cases underwent brain autopsies and demonstrated marked astrogliosis in the thalamus and neuron loss in various brain regions [14]. T188K gCJD is the second most common form of gCJD in China. Rapidly progressive dementia and mental symptoms appeared in most of the cases at the time of disease onset and cerebellar disorders and walking instability were also described among the initial symptoms. With the development of disease, dementia, visual or cerebella disturbance and pyramidal or extrapyramidal dysfunction appeared in over 80% of the cases. Myoclonus and akinetic mutism appeared in over half of the cases. E200K gCJD cases were more abundant than sCJD, in which dementia and additional neurological symptoms were commonly observed.

**Table 1:** The clinical manifestations of various human genetic prion diseases.

	Case No	dementia	myoclonus	visual or cerebella disturbance	pyramidal or extrapyramidal dysfunction	akinetic mutism
1 OR	1	1	1	-	1	-
7 ORs	1	1	1	1	1	-
P102L	3	3	1	1	3	1
G114V	2	2	0	1	1	0
R148H	1	1	1	1	1	1
D178N	27	19 (70.37%)	14 (51.85%)	14 (51.85%)	14 (51.85%)	3 (11.11%)
V180I	1	1	1	-	1	-
T183A	1	1	-	-	-	-
T188K	16	13 (81.25%)	9 (56.25%)	14 (87.50%)	14 (87.50%)	7 (43.75%)
E196A	2	2	1	2	2	1
E196K	1	1	1	-	1	-
E200K	9	8 (88.89%)	6 (66.67%)	5 (55.56%)	8 (88.89%)	7 (77.78%)
E200A	1	1	1	1	1	-
V203I	1	1	1	1	-	-
R208H	2	2	1	2	1	1
Total	69	57 (82.61%)	39 (56.52%)	43 (63.32%)	49 (71.01%)	21 (30.43%)

A disease-related family history of each patient was carefully collected by the local clinical neurologists and the staff of the CJD surveillance center. Analysis of these histories indicated that 43.48% of patients (30 of 69) had family members in their parents' or grandparents' generations who exhibited similar neurological disorders or manifestations, including the case with 7-ORs insertion, one (out of three) P102L case, two G114V cases, the R148H case, 16 (out of 27) FFI cases, the T183A case, six (out of 16) T188K cases, one (out of 9) E200K case and one (out of two) R208H case (Table 2). The case with a 1-OR insertion, the V180I case, two E196A cases, the E196K case, the E200A case and the V203I case did not record this family relationship (Table 2). The number of patients with a family history of the condition is similar with the rate of incidence in Europe [17]. This study assumes that patients with mutations in the N-terminal segment might be more likely to have family problems. When compared with the data of the T188K (37.50%) and E200K (11.11%) groups, more FFI cases (59.26%) exhibited this disease-related family history. Additionally, we asked all families with disease-related family histories to perform *PRNP* gene assays for their family members. Family members from 23 families agreed to donate blood samples. As shown in Table 2, the same mutations within the *PRNP* gene that were observed in the indexed patients were also identified in at least one family member of all tested families.

**Table 2:** Family histories and *PRNP* sequencing results of various human genetic prion diseases.

	Case No	Family history		*PRNP* mutation in family members
		yes	no	
1 OR	1	-	1	-
7 ORs	1	1	-	1 ND
P102L	3	1	2	1 family
G114V	2	2	-	2 families
R148H	1	1	-	1 family
D178N	27	16 (59.26%)	11 (40.74%)	12 families; 4 ND
V180I	1	-	1	-
T183A	1	1		1 ND
T188K	16	6 (37.50%)	10 (62.50%)	5 families; 1 ND
E196A	2	-	2	-
E196K	1	-	1	-
E200K	9	1 (11.11%)	8 (88.89%)	1 family
E200A	1	-	1	-
V203I	1	-	1	-
R208H	2	1	1	1 family
Total	69	30 (43.48%)	39 (56.52%)	23 families; 7ND

### The clinical examinations and laboratory tests for Chinese genetic prion diseases

Out of 69 patients, only five cases had postmortem brain tissue collected: the case with 7-ORs insertion [17], three FFI cases [12,13,14] and one G114V gCJD case [15,16,18], whose neuropathological changes and the presence of PrP^Sc^ were previously described. Sixty-five (94.2%), 61 (90.41%) and 55 (79.71%) cases underwent CSF 14-3-3 tests, MRI recordings and EEG examinations, respectively. All 69 cases underwent at least one of these three examinations. The results of CSF 14-3-3 tests as well as of MRI and EEG examinations of these 69 cases are summarized in Table 3 In the assay of CSF 14-3-3, all tested cases with point-mutations (i.e., P102L, G114V, R148H) at the N-terminal segment as well as a majority of the FFI cases (62.96%) were negative. Meanwhile, those with mutations at the C-terminus (E196A, E200K, E200A, V203I and R208H) as well as most of the T188K gCJD cases (68.75%) were positive. sCJD-associated abnormalities in the MRI recordings were much more frequently observed in the T188K (68.75%) and E200K (77.78%) groups but were very rare in the FFI cases (1/27; 3.7%). In these genetic prion diseases, MRI abnormalities were seen in the patients with 7-ORs, one tested P102L case, the V180I case, two E196K cases and the E200A case. PSWCs in EEG were detected in 66.67% of E200K cases, but only in 12.50% of T188K cases. As expected, none of the tested FFI cases showed PSWCs in EEG examinations. EEG abnormalities were also recorded in the patients with 7-ORs, two tested P102L cases, the V180I case, the E196A case and the V203I case.

**Table 3:** CSF 14-3-3, MRI and EEG status in various human genetic prion diseases.

	Case No.	CSF 14-3-3			MRI			EEG		
		Pos	Neg	ND	Pos	Neg	ND	Pos	Neg	ND
1 OR	1	1	-	-	-	1	-	-	-	1
7 ORs	1	-	-	1	1	-	-	1	-	-
P102L	3	-	2	1	1	-	2	2	-	1
G114V	2	-	2	-	-	2	-	-	1	1
R148H	1	-	1	-	-	1	-	-	1	-
D178N	27	9 (33.33%)	17 (62.96%)	1 (3.7%)	1 (3.7%)	21 (77.78%)	5 (18.52%)	0 (0.0%)	24 (88.89%)	3 (11.11%)
V180I	1	-	1	-	1	-	-	1	-	-
T183A	1	-	1	-	-	1	-	-	-	1
T188K	16	11 (68.75%)	4 (25.00%)	1 (6.26%)	11 (68.75%)	5 (31.25%)	0 (0.0%)	2 (12.50%)	12 (75.00%)	2 (12.50%)
E196A	2	2	-	-	2	-	-	1	-	1
E196K	1	-	1	-	-	1	-	-	1	-
E200K	9	9 (100%)	0 (0.0%)	0 (0.0%)	7 (77.78%)	2 (22.22%)	0 (0.0%)	6 (66.67)%	1 (11.11%)	2 (22.22%)
E200A	1	1	-	-	1	-	-	-	1	-
V203I	1	1	-	-	-	1	-	1	-	-
R208H	2	1	1	-	-	1	1	-	-	2
Total	69	35 (50.72%)	30 (43.48%)	4 (5.80%)	25 (36.23%)	36 (52.17%)	8 (11.59%)	14 (20.29%)	41 (59.42%)	14 (20.29%)

### Intervals between disease onset and neurologist visit, diagnosis and disease outcome in Chinese patients with genetic prion diseases

To understand disease progression and persistence, the time intervals between disease onset and the first neurologist visit, the final diagnosis and the death of the patient were individually assessed and the data are listed in Table 4. Among the 69 patients, 40 cases had definitive death times, 15 cases resulted in a loss of contact after diagnosis or during follow-up, and the remaining 14 patients were still alive by the end of Sept 2014. Although the median times of the intervals from onset to neurologist visit, to final diagnosis and to death of the 69 patients were 92, 153 and 252 days, respectively, the actual interval times differed considerably across various diseases and across individuals with the same diseases. The patients with an extra octarepeat insertion demonstrated longer intervals for diagnosis and a longer clinical course, despite having lost contact with the patient whose disease included an insertion more than 3 years after onset. The cases with the point-mutations in the region of the N-terminus (P102L, G114V and R148H) appeared to require more time for diagnosis and enjoyed a longer survival time than those with the mutations in the C-terminus; however, the number of cases in the diseases types are limited.

**Table 4:** The time interval between disease onset and the initial neurologist visit, diagnosis and death of various human genetic prion disease patients.

	Case No	From onset to neurologist visit	From onset to diagnosis	From onset to death	Remark*
		Median (range) (day)	Median (range) (day)	Median (range) (day)	
1 OR	1	1034	1068	-	lost
7 ORs	1	730	1461	1507	-
P102L	3	287 (59–1161)	658 (140–1123)	804 (303–1305) (n = 2)	1 lost
G114V	2	281 (17–546)	354 (156–552)	760 (n = 1)	1 lost
R148H	1	111	180	-	alive
D178N	27	170 (25–775)	409 (91–820)	293 (123–945) (n = 16)	7 lost; 5 alive
V180I	1	13	112	-	alive
T183A	1	7	25	-	alive
T188K	16	69 (19–167)	158 (48–246)	122 (69–274) (n = 11)	2 lost; 3 alive
E196A	2	79 (9–129)	96 (29–137)	381 (516–731)	-
E196K	1	9	29	516	-
E200K	9	71 (14–167)	113 (73–275)	285 (135–409) (n = 4)	3 lost 2 alive
E200A	1	29	71	61	-
V203I	1	14	39	274	-
R208H	2	98 (44–151)	145 (84–206)	107 (n = 1)	1 alive
Total	69	92	153	252 (n = 40)	15 lost; 14 alive

*: "lost" indicates that contact with the patient was lost after diagnosis or during follow-up; "alive" indicates the patients who were still alive at the end of Sept 2014.

Furthermore, the differences in the time intervals between onset and neurologist visit, final diagnosis and death among the FFI, T188K and E200K gCJD groups that possessed sufficient cases for statistical analysis were individually analyzed. When compared with those of the T188K and E200K gCJD groups, the patients of FFI presented a wider range of time in the intervals between disease onset and neurologist visit, final diagnosis and death (S1 Fig). Significantly longer intervals between onset and neurologist visit and final diagnosis of FFI cases (P<0.05) might indicate a relatively slow progression and indistinguishable clinical manifestation at the early stage. The time interval between disease onset to death of the FFI cases was significantly longer than that of the T188K cases (P<0.01) but comparable with the time interval in the E200K case. The cases in the T188K gCJD group demonstrated similar intervals between onset and neurologist visit and final diagnosis as, but shorter intervals from onset to death (P<0.05) than, the E200K cases. This may indicate a more rapid progression and shorter survival time of T188K gCJD.

To assess the potential differences in the difficulty of diagnosis and relevant influence factors among various genetic prion diseases, the time between neurologist visit and the final diagnosis of each patient was analyzed. The interval times varied largely from one week to more than two years, with a median of 45 days. Because of the limited number of cases in the many subtypes of prion diseases, the patients were grouped as FFI, T188K, E200K and others. The FFI and T188K groups demonstrated slightly long and short median interval times, but without significant differences among these four groups. These patients were further grouped based on the year of diagnosis, the age at onset, and the location of residency. This allowed for us to screen for factors that influence the interval times. No significant differences were observed among the groups based on the surveillance year between 2006 and 2014, the age at which patients presented clinical manifestations, or the location of residency (S2 Fig).

## Discussion

Between 2006 and Sept 2014, 69 cases of 15 different genetic prion diseases were diagnosed genetically and/or pathologically via the China National Surveillance Program for CJD. This is the first and largest systematic report concerning genetic prion diseases in China. Except one Miao-Chinese, all cases included Han-Chinese patients. In China, there are 56 nationalities. Han nationality is the majority of the population and accounts for 90.56% of the population. Miao nationality is the fourth largest of the national minorities and whose members reside mostly in the Southwestern provinces of China. The total population of Miao-Chinese is approximately 9 million. Similar to the Han-Chinese [7], the polymorphisms of codon 129 of all cases of genetic prion diseases are the Met/Met homozygote. FFI, T188K and E200K gCJD are the most frequently identified genetic prion diseases, which account for approximately 75% of all cases. Three cases with the P102L mutation and that are associated with GSS have been detected in the past eight years and no other GSS-associated mutation has been observed. The remaining cases are rare *PRNP* mutations, which account for one to two percent of gCJD cases. In agreement with the idea that genetic CJD accounts for 5–15% of all CJD cases, Chinese genetic prion diseases account for 6–12% (median 8.7%) of all diagnosed CJD cases each surveillance year. More than 56% of cases do not have clear disease family histories. The age at disease onset of the patients with genetic prion diseases is approximately 10 years younger than that of sCJD cases [7]. These data highlight that the pattern of Chinese genetic prion diseases possesses common features of genetic prion diseases worldwide [19,20,21,22,23,24], though subtle differences remain across disease sub-types.

The geographical distribution of the cases of genetic prion diseases seems to be dispersed across mainland China; however, 20 cases are from one province (Henan) and consists of six different diseases, including 13 FFI cases. Analysis of these 20 cases did not identify any genetic relationship among families, except in two FFI cases, which originated from one family. The Henan province is the most populous province in China, containing approximately 100 million residents. The CJD surveillance in the Henan province is also the most active, with more reported suspected CJD cases each year. This may partially account for this geographical pattern; however, the total number of genetic prion diseases and the involved provinces from the northern part of China (bounded by the Yangtze river) are extensive when compared to the southern part of China. Whether such disease distribution features are linked to the large migrations of the Han Chinese in the past requires further analysis.

The clear predominance of FFI (39%), T188K (23%) and E200K (13%) cases in Chinese genetic prion diseases may illustrate a unique pattern that differs from not only Caucasian but also North-east Asian (e.g., Japanese and Korean) disease trends [4,25,26]. The literature indicates that E200K is the most common mutation in many European countries [22,27], while FFI and various GSS mutations are also frequently observed [19,28,29,30]. In some special regions, such as the Italo-Greek villages of the southeast Calabria region in Italy [31], the Slovakian [32] and the Libyan Jews in Israel [33], the distributions of the E200K mutation, either in patients or in the health carriers, are extremely elevated. E200K gCJD is also the most common genetic prion disease in Argentina, which accounts for 15.6% of all reported human prion diseases between 1997 and 2008 [22]. In Japan, the most common mutation in *PRNP* is V180I (41.2%). Other frequent mutations include P102L (18.1%), E200K (17.1%) and M232R (15.3%) [34]. In Korea, the V180I, E200K, P102L, V203I, and M232R mutations have each been reported in more than two clinical cases [4]. When compared with Japanese and Korean cases, the prevalence of the E200K mutation in Chinese genetic prion diseases appears similar. Yet, the number of FFI and T188K gCJD cases are markedly more increased while P105L, V180I and V232R gCJD cases are rare or even undetected.

The clinical signs and clinical examinations among those 15 genetic prion diseases widely vary. As expected, the number of Chinese FFI cases displays a distinct pattern among the gCJD cases, with fewer clinical manifestations being described for sCJD. PSWCs in EEG are not detected in all FFI cases and MRI abnormalities have been observed in only one FFI case. CSF 14-3-3 is positive in approximately a third of FFI cases. Conversely, considerably greater portions of patients with T188K and E200K demonstrate abnormalities in these three examinations, thereby indicating again the significance of CSF-14-3-3, MRI and EEG in some types of gCJD.

Despite the limited patient numbers for many subtypes of gCJD in this study, we noticed some unique patterns regarding different locations of the disease-associated mutations. The diseases that are associated with mutations in the N-terminus (i.e., before aa 183) of the PrP protein are associated with an earlier age of onset of the disease symptoms, are negative in CSF-14-3-3, are frequently associated with a family history and have longer time intervals between diagnosis of the disease and completion of its clinical course. Conversely, the patients with mutations in the C-terminus of the PrP have relatively high ratios of CSF-14-3-3 positivity and short intervals for disease recognition and clinical course. Meanwhile, the age of onset is older, and fewer patients demonstrate a family history of the disease. The relevant molecular mechanisms remain unclear. Two Chinese patients with extra octarepeat insertions in this study appear to have longer disease durations. For example, disease duration is more than four years in the cases with 7-Ors [10]. Other reported cases with extra octarepeat insertions also present long durations, being 16 years in one case [35,36,37]. Unfortunately, the case with 1-OR insertion went without contact after eight months following diagnosis. The known duration of this case is already 43 months at the last follow-up.

T188K gCJD is the second most common genetic prion disease in the Han Chinese population, which has been described only in three German and one Austrian previously [38,39]. Surprisingly, the T188K mutation has not reported in Japanese and Korean patients in the literature so far, which reflects the diversity in genetics among the East Asian races. The onset ages of T188K gCJD cases are older than those of FFI case, but similar to the E200K case. Although the time intervals between disease onset to neurologist visit and to final diagnosis are comparable between the cases of T188K and E200K, the clinical courses of T188K patients are markedly shorter. The durations of the other four T188K gCJD Caucasian cases, whose genotypes of codon 129 are Met/Val heterozygote, are also relatively short (ranging from 5 to 13 months) [38]. Meanwhile, an older age at onset, a high ratio of MRI abnormality, fewer cases with a family history of the disease and a short survival time may constitute the features of T188K gCJD. Like the characteristics of FFI in many other studies, Chinese FFI cases also demonstrate relatively long clinical durations. The time until diagnosis of FFI cases is also longer than in T188K and E200K gCJD, which may indicate a relatively slow progression and a more atypical clinical appearance at early stages.

We have also analyzed the time intervals between the initial neurologist visit and the final diagnosis and the factors that possibly influence this interval in the 69 patients. Because all suspected CJD cases that are reported to the CJD Surveillance Center include *PRNP* sequencing assays, the time interval from neurologist visit to final diagnosis fairly reflects the recognition times by neurologists. Although the individual interval of the patient varies considerably, with the average and median times being 71.1 and 45 days, respectively, there is no large difference among the patients in the FFI, T188K, E200K and other mutation groups. Other factors, such as the surveillance year, age at onset and place of residence of the patients (which may represent the economic status of the patient) are also unrelated to disease recognition time. Further analysis revealed that the patients with longer intervals between the initial neurologist visit and final diagnosis usually have had multiple hospital transfers between small clinics and large polyclinics. In another study, we proposed that the diagnosed CJD patients from the Beijing region have fewer hospital transfers and a shorter time interval between disease onset and the final diagnosis than do those outside of the Beijing region [40], which implies that clinician knowledge of prions and prion diseases is essential for shortening the diagnostic time for CJD.

## Supporting Information

S1 Fig Comparison of the intervals between onset and neurologist visit. A. Final diagnosis. B. Death of the patients of FFI, T188K and E200K gCJD. The P values are indicated above.
(TIF)

S2 Fig Analysis of the intervals from neurologist visit to final diagnosis based on the different diseases. A. The surveillance year. B. The age of onset. C. The residence of patients. D. The P values are indicated.
(TIF)
