# Supplementary figures and images for: The Features of Genetic Prion Diseases Based on Chinese Surveillance Program
Source: PLoS One. 2015 Oct 21;10(10):e0139552. doi: 10.1371/journal.pone.0139552 (PMC4619501; doi:10.1371/journal.pone.0139552)

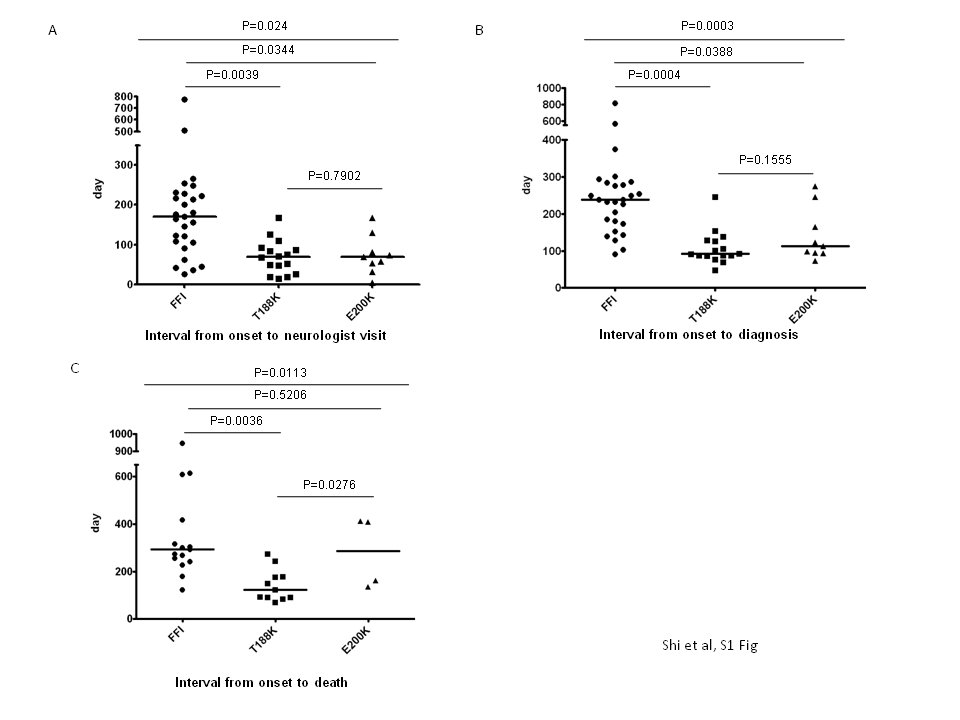

Supplement: S1 Fig — A. Final diagnosis. B. Death of the patients of FFI, T188K and E200K gCJD. The P values are indicated above. (TIF) [file pone.0139552.s001.TIF]

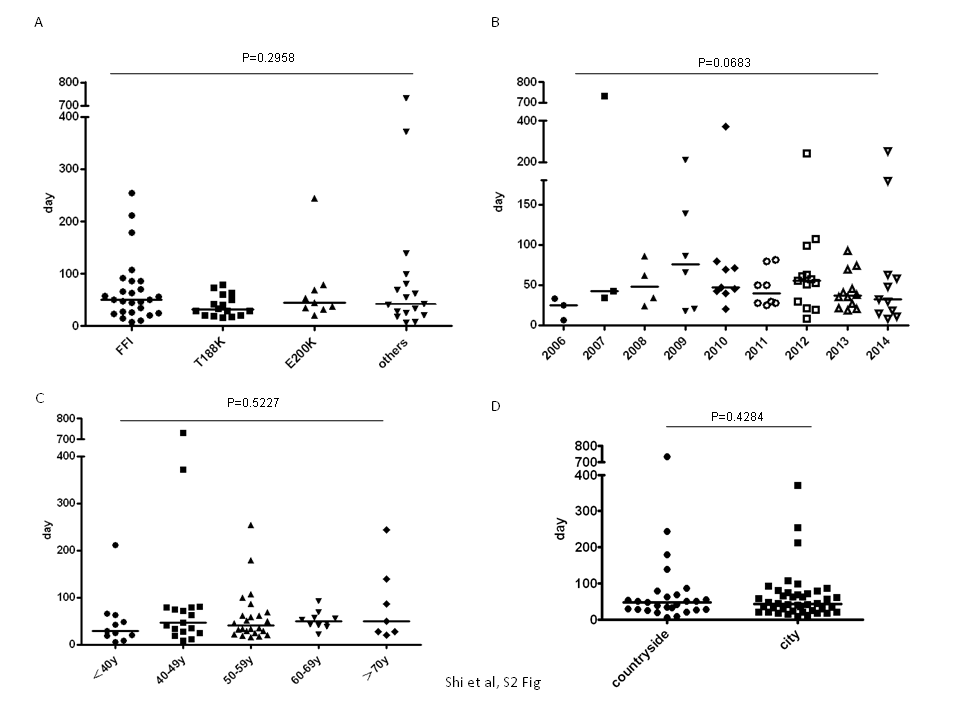

Supplement: S2 Fig — A. The surveillance year. B. The age of onset. C. The residence of patients. D. The P values are indicated. (TIF) [file pone.0139552.s002.TIF]
